# A Case of Immunoglobulin G4-Related Gastrointestinal Disease Diagnosed From Persistent Diarrhea and Abdominal Pain

**DOI:** 10.1016/j.gastha.2023.07.009

**Published:** 2023-07-21

**Authors:** Takehiro Hirano, Yujiro Kawakami, Sayaka Nakabayashi, Kohei Wagatsuma, Keisuke Ishigami, Yoshiharu Masaki, Ayako Murota, Masatoshi Kanda, Shintaro Sugita, Kenji Notohara, Hiroshi Nakase

**Affiliations:** 1 Department of Gastroenterology and Hepatology, Sapporo Medical University School of Medicine, Sapporo, Hokkaido, Japan; 2 Sapporo Medical University School of Medicine, Sapporo, Hokkaido, Japan; 3 Department of Rheumatology and Clinical Immunology, Sapporo Medical University School of Medicine, Sapporo, Hokkaido, Japan; 4 Department of Surgical Pathology, Sapporo Medical University School of Medicine, Sapporo, Hokkaido, Japan; 5 Department of Anatomic Pathology, Kurashiki Central Hospital, Kurashiki, Okayama, Japan

**Keywords:** IgG4-Related Disease, IgG4-Related Gastrointestinal Disease, IgG4-Related Dacryoadenitis and Sialadenitis, Diarrhea, Abdominal Pain, Inflammatory Disease

## Abstract

Immunoglobulin G4-related disease (IgG4-RD) is a systemic inflammatory disease characterized by the infiltration of IgG4-positive plasma cells and fibrosis in organs throughout the body. IgG4-RD involvement in the gastrointestinal (GI) tract (IgG4-related GI disease; IgG4-GID) is rare, and the disease concept remains unclear. Generally, IgG4-GID has been reported with morphological changes, including ulcers, strictures, and submucosal tumors. Here, we report a case of IgG4-GID with persistent diarrhea and abdominal pain in which typical endoscopic findings were absent. This case suggests the unidentified clinical features of IgG4-GID.

## Introduction

Immunoglobulin G4 (IgG4)-related disease (IgG4-RD) is a systemic inflammatory disease characterized by increased IgG4 in serum, as well as infiltration of IgG4-positive plasma cells and fibrosis in various organs throughout the body.^1^ Although the detailed pathophysiology of IgG4-RD has yet to be elucidated, the responsiveness to immunosuppression suggests an autoimmune etiology.^2^ The consensus statement on the treatment for IgG4-RD states that glucocorticoids are the first-line agents.^3^ The salivary glands, lungs, and pancreas have been known to be the predilection sites in IgG4-RD. Additionally, lesions in the gastrointestinal (GI) tract have also been reported (IgG4-related GI disease; IgG4-GID).^4^ IgG4-GID has been reported with endoscopic findings, such as ulcers, strictures, and submucosal tumors. However, the etiology remains unclear.

## Case Report

**Figure 1:**
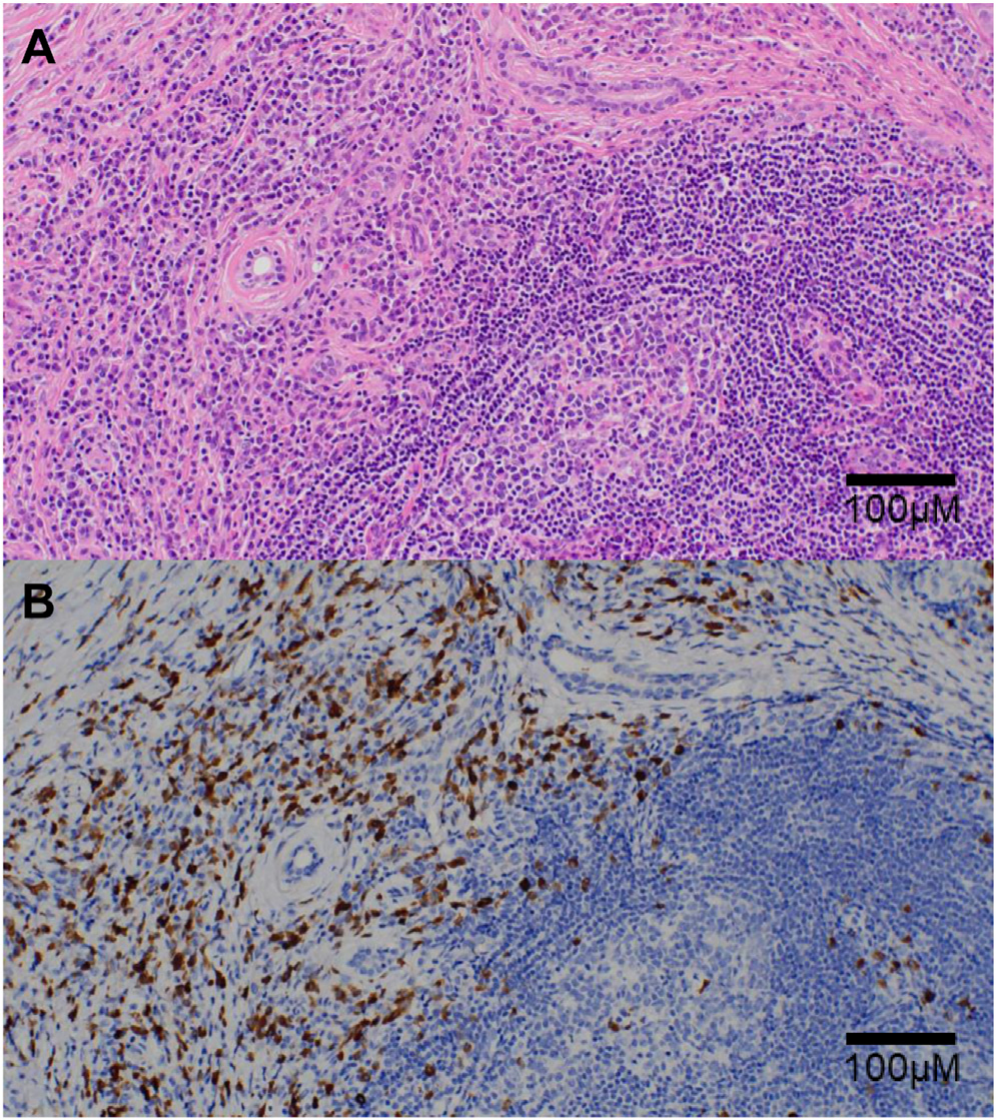
Histopathological findings in the submandibular gland. (A) Hematoxylin–eosin staining and (B) IgG4 immunohistochemistry. Magnification: 200×. Scale bars are shown in the figure.

**Figure 2:**
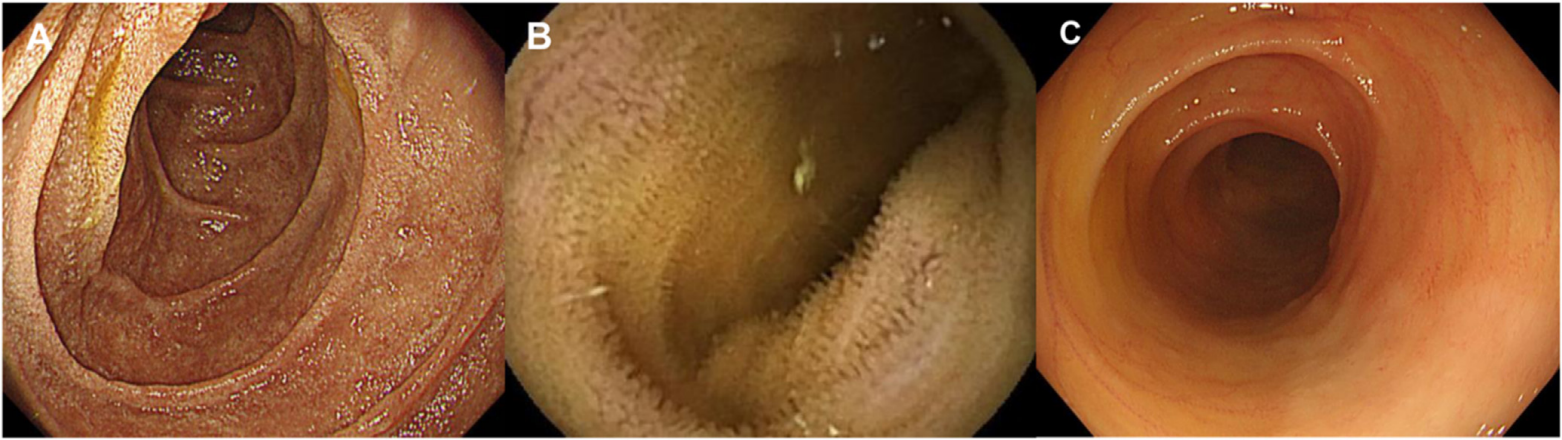
Representative endoscopic findings of the gastrointestinal tract before the treatment of IgG4-RD. (A) Esophagogastroduodenoscopic findings in the duodenum. (B) Capsule endoscopic findings in the ileum. (C) Colonoscopic findings in the terminal ileum. These examinations did not reveal any morphological changes such as ulcers, strictures, or submucosal tumors, which are frequently reported in IgG4-related gastrointestinal diseases.

**Figure 3:**
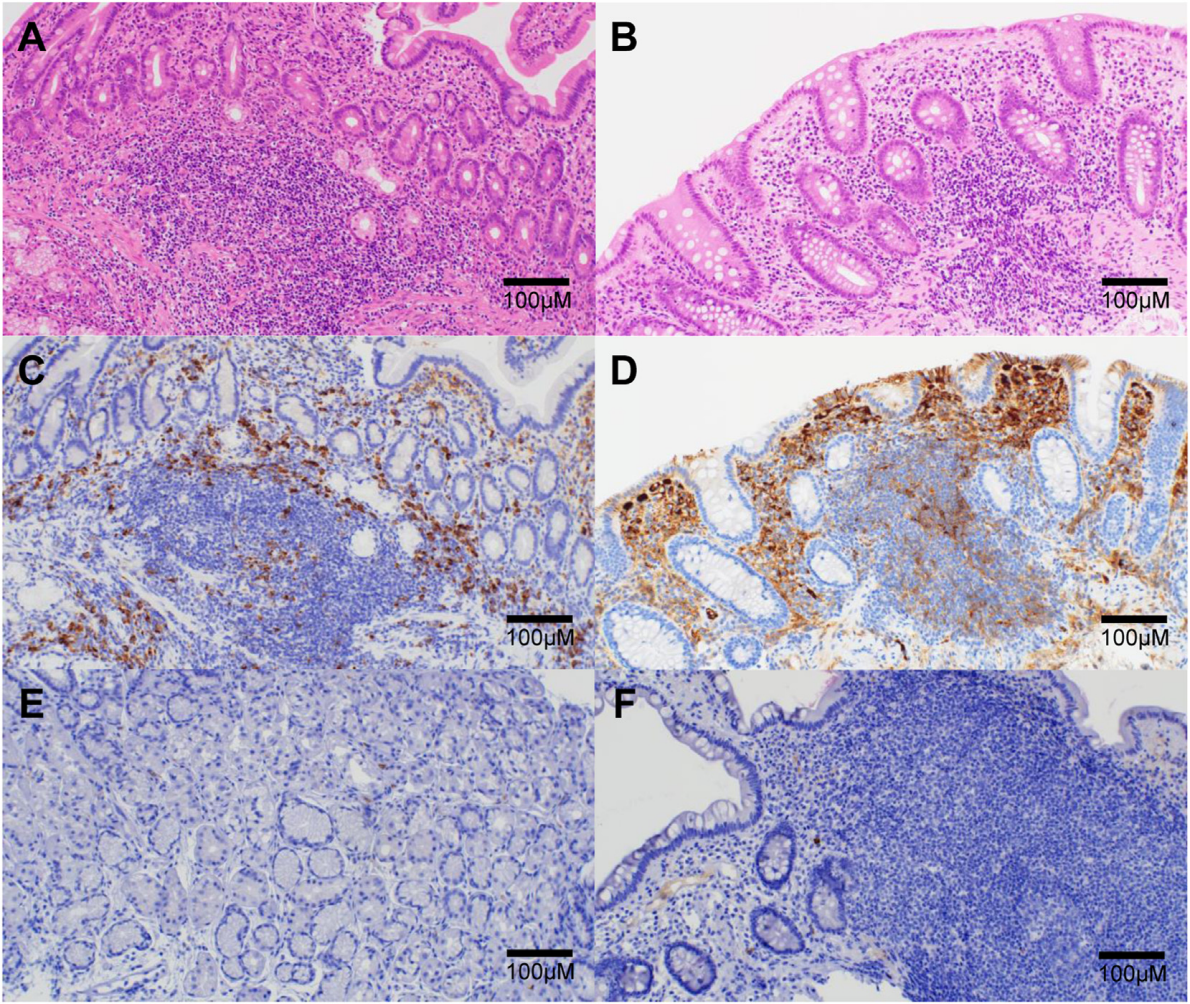
Histopathological findings in the duodenum and terminal ileum. The upper row shows the hematoxylin–eosin staining of the duodenum (A) and terminal ileum (B). The middle row shows IgG4 immunohistochemistry (IHC) of the duodenum (C) and terminal ileum (D) before treatment. The lower row shows the IgG4 IHC of the duodenum (E) and terminal ileum (F) after PSL treatment. Magnification: 200×. Scale bars are shown in the figure.

**Figure 4:**
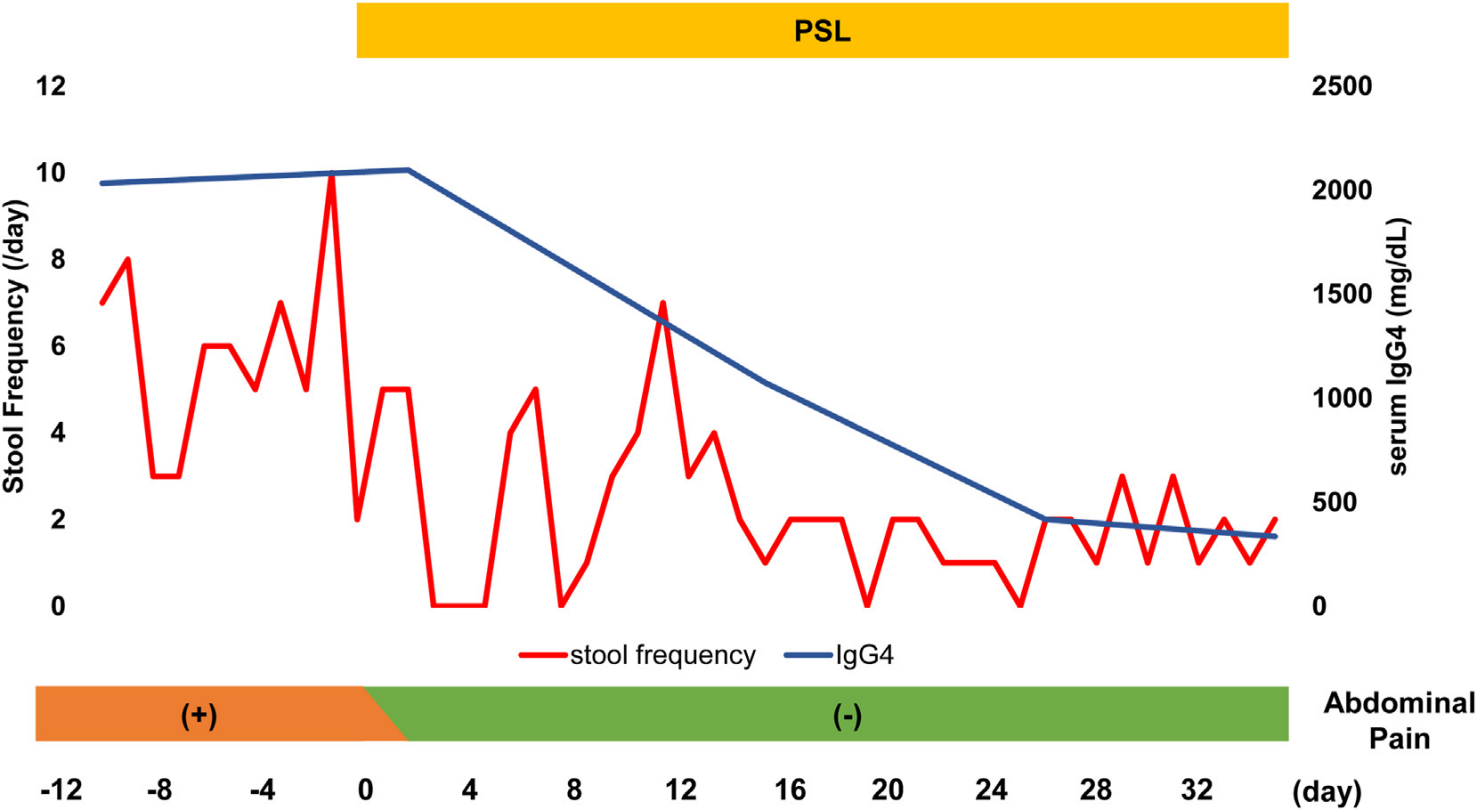
The clinical course of this case (stool frequency, serum IgG4 levels, and abdominal pain).

A 72-year-old woman with high serum IgG levels and enlarged abdominal lymph nodes on computed tomography (CT) was referred to our hospital. Serological tests revealed markedly elevated serum IgG (5563 mg/dL) and IgG4 (2670 mg/dL) levels. Contrast-enhanced CT revealed swelling in the bilateral submandibular gland and diffuse enlargement of the pancreas. There were no notable findings in the GI tract with the CT. We performed a biopsy of the submandibular lesion, which was consistent with the pathological features of comprehensive diagnostic criteria (dense lymphocyte and plasma cell infiltration with fibrosis, ratio of IgG4-positive plasma cells /IgG-positive cells greater than 40%, and the number of IgG4-positive plasma cells greater than 10 per high-powered field),^5^ and diagnosed the patient with IgG4-RD (Figure 1). On the other hand, she has suffered from intermittent abdominal pain and chronic diarrhea approximately ten times daily. These symptoms have persisted for more than six months. We performed esophagogastroduodenoscopy, capsule endoscopy, and colonoscopy, which did not reveal any organic lesions that could have caused her abdominal symptoms (Figure 2). However, histopathological evaluation of biopsy specimens of the duodenum and terminal ileum revealed an inflammatory cell infiltration composed mainly of lymphocytes and plasma cells (Figure 3A and B) and IgG4-positive cell infiltration deep into the lamina propria (Figure 3C and D). Based on these pathological findings (ratio of IgG4-positive plasma cells /IgG-positive cells greater than 40%, the number of IgG4-positive plasma cells greater than 10 per high-powered field, and bottom-heavy plasmacytosis^4^), we diagnosed the patient with IgG4-GID. We started the IgG4-RD treatment by administering 30 mg of prednisolone (PSL) per day and tapering it to 5 mg per day. Her abdominal pain and diarrhea improved immediately after the initiation of PSL (Figure 4). Serum IgG4 levels decreased considerably, and CT after seven weeks of treatment indicated shrinkage of the submandibular gland and pancreas. We did not detect lymphocyte or IgG4-positive plasma cell infiltration in endoscopic biopsy specimens of the duodenum and terminal ileum after six months of treatment (Figure 3E and F). The patient received maintenance treatment with 5 mg of PSL for two years, and there was no relapse in serological tests, imaging, or abdominal symptoms.

## Discussion

We experienced a case of IgG4-RD wherein the GI mucosa appeared normal with no submucosal tumor or ulcerative changes; however, the pathology of biopsy specimens from the GI mucosa was consistent with the pathological features of IgG4-RD comprehensive diagnostic criteria.^5^ The patient had abdominal symptoms, such as diarrhea and abdominal pain. Of note, after the initiation of PSL, the symptoms improved with the disappearance of IgG4-positive cells in the GI mucosa. Thus, the present case is interesting in the context of IgG4-RD pathogenesis.

In a systematic review of 42 cases of IgG4-GID, submucosal tumors (25/42, 59.5%) and ulcers/erosions (19/42, 45.2%) were frequent in the esophagogastroduodenoscopy findings of IgG4-GID.^6^ Abdominal pain was the most common abdominal symptom of IgG4-GID (12/40, 30.0%), whereas diarrhea was reported in only one case.^6^ However, there have been no observational studies of biopsy samples from endoscopically normal-appearing GI mucosa in patients diagnosed with IgG4-RD. In this case, mucosal biopsy specimens of the duodenum and ileum, which appeared normal, showed IgG4-positive plasma cell infiltration and bottom-heavy plasmacytosis. These results suggest that the IgG4-positive cells do not necessarily form lesions at the site of infiltration but may infiltrate organs throughout the body. It may also explain why IgG4-RD has ectopic and heterochronic pathogenesis; several autoantigens, such as prohibitin,^7^ galectin-3,^8^ and laminin 511^9^ have been reported to involve in the pathogenesis of IgG4-RD, and these are not specific to the pancreas or salivary glands. Therefore, it is not surprising that IgG4-positive cells can infiltrate the GI tract. The pathogenesis of IgG4-RD, including the presentation of these antigens to naive T and B cells, still needs to be clarified. However, the presence of IgG4-GID, including our case, may suggest that an abnormal immune response in the GI tract may result in disease development.

It has been reported that neurotransmitters from mast cells, such as histamine and serotonin, and inflammatory cytokines from immune cells induce intestinal dysmotility.^10^^,^^11^ The patient’s abdominal pain and diarrhea subsided immediately after PSL was initiated. This indicated that the infiltration of immune cells into the intestinal mucosa can cause abdominal symptoms. However, we could not fully prove the involvement of IgG4-positive plasma cell infiltration in the small bowel because we performed a biopsy of only the duodenum and the terminal ileum.

In conclusion, IgG4-RD involves the GI tract even in cases without endoscopic findings and may induce abdominal symptoms. GI biopsies in IgG4-RD patients with various clinical features may contribute to a better understanding of the clinical features in IgG4-GID.
